# High CD4 counts associated with better economic outcomes for HIV-positive adults and their HIV-negative household members in the SEARCH Trial

**DOI:** 10.1371/journal.pone.0198912

**Published:** 2018-06-26

**Authors:** Aleksandra Jakubowski, Katherine Snyman, Dalsone Kwarisiima, Norton Sang, Rachel Burger, Laura Balzer, Tamara Clark, Gabriel Chamie, Starley Shade, Craig Cohen, Elizabeth Bukusi, Edwin Charlebois, Moses Kamya, Maya Petersen, Diane Havlir, Harsha Thirumurthy

**Affiliations:** 1 School of Medicine, Stanford University, Stanford, CA, United States of America; 2 School of Medicine, University of California, San Francisco, CA, United States of America; 3 Infectious Diseases Research Collaboration, Kampala, Uganda; 4 Kenya Medical Research Institute, Nairobi, Kenya; 5 School of Public Health and Health Sciences, University of Massachusetts, Amherst, United States of America; 6 Makerere University, Kampala, Uganda; 7 School of Public Health, University of California, Berkeley, CA, United States of America; 8 Perelman School of Medicine, University of Pennsylvania, Philadelphia, PA, United States of America; Boston University School of Public Health, UNITED STATES

## Abstract

**Background:**

Country decisions to scale-up “test and treat” approaches for HIV depend on consideration of both the health and economic consequences of such investments. Evidence about economic impacts of expanded antiretroviral therapy (ART) provision is particularly relevant for decisions regarding foreign assistance levels for HIV/AIDS programs. We used baseline data from the Sustainable East Africa Research in Community Health (SEARCH) cluster randomized controlled trial in Kenya and Uganda to examine the association between HIV status, CD4+ T-cell counts, viral suppression, and multiple indicators of economic well-being.

**Methods and findings:**

Socio-economic surveys were conducted in households with HIV-positive and HIV-negative adults sampled after a census of 32 communities participating in the SEARCH trial (NCT01864603). Data were obtained for 11,500 individuals from 5,884 households in study communities. Participants were stratified based on their own HIV status as well as CD4 counts and viral suppression status if they were HIV-positive. HIV-negative participants residing in households with no HIV-positive adults were considered separately from HIV-negative participants residing in households with ≥1 HIV-positive adult. Generalized estimating equation models were used to examine the relationship between HIV status, CD4 counts, ART, viral suppression, and outcomes of employment, self-reported illness, lost time from usual activities due to illness, healthcare utilization, health expenditures, and hospitalizations. In all models, HIV-negative participants in households with no HIV-positive persons were the reference group.

There was no significant difference in the probability of being employed between HIV-positive participants with CD4>500 and the reference group of HIV-negative participants residing in households with no HIV-positive adults (marginal effect, ME, 1.49 percentage points; 95% confidence interval, CI, -1.09, 4.08). However, HIV-positive participants with CD4 351–500 were less likely to be employed than the reference group (ME -4.50, 95% CI -7.99, -1.01), as were HIV-positive participants with CD4 ≤350 (ME -7.41, 95% CI -10.96, -3.85). Similarly, there was no significant difference in employment likelihood between HIV-negative participants who resided in households with a CD4>500 HIV-positive person and the reference group (ME -1.78, 95% CI -5.16, 1.59). HIV-negative participants residing with an HIV-positive person with CD4 351–500, however, were less likely to be employed than the reference group (ME -7.03, 95% CI -11.49, -2.57), as were people residing with a household member with CD4 ≤350 (ME -6.28, 95% CI -10.76, -1.80). HIV-positive participants in all CD4 categories were more likely to have lost time from usual activities due to illness and have incurred healthcare expenditures. Those with CD4>500 had better economic outcomes than those with CD4 351–500, even among those not virally suppressed (*p* = 0.004) and not on ART (*p* = 0.01).

**Conclusions:**

Data from a large population-representative sample of households in east Africa showed a strong association between the health of HIV-positive persons and economic outcomes. The findings suggest there may be economic benefits associated with maintaining high CD4 counts, both for HIV-positive persons and their HIV-negative household members. The association of high CD4 counts with improved outcomes is consistent with the hypothesis that early ART initiation can avert declines in employment and other economic outcomes. Prospective longitudinal evaluation is needed to assess the causal impact of early ART initiation on economic functioning of households.

## Introduction

Compelling evidence on the public health benefits of antiretroviral (ART) treatment as prevention and the individual-level benefits of early ART initiation [1, 2] was the foundation for the 2015 WHO guidelines that recommend treatment for all persons living with HIV. Given the promise ART now holds for HIV-positive people and the broader population, the UNAIDS established the ‘90-90-90 targets’ that call for 90% coverage of HIV testing, ART initiation, and treatment adherence among HIV-positive persons [3, 4]. Despite these data and recommendations, less than half of countries have adopted the WHO guidelines and pace of adaptation is threatened even among countries moving forward to “HIV test and treat” [5]. Despite the strong public health case and economic rationale, a number of factors including resource constraints have contributed to these delays [3, 6–8]. Uncertainty about levels of support from the US government for HIV/AIDS programs through the President’s Emergency Plan For AIDS Relief (PEPFAR) and other initiatives [9] could put an even greater financial burden on low-income countries that depend on this financing. Policymakers could therefore benefit from assessments of the potential economic gains of finding and keeping HIV-positive persons in the high CD4 range through early ART initiation—gains that may be realized by the affected HIV-positive individual and their household members.

Longitudinal studies conducted in sub-Saharan Africa (SSA) during the past decade show that ART initiation among individuals with low CD4+ T-cell counts, in line with prior WHO guidelines, leads to dramatically improved individual- and household-level economic outcomes [10–18]. These studies report sustained improvements in outcomes ranging from labor force participation, income, and quality of life for the individuals receiving ART. A limited number of additional studies have also documented benefits to household members of treated adults, particularly in the form of reduced domestic labor burden [19] and increased school enrollment for children [20]. One study also showed that ART expansion led to increased work hours of HIV-negative people whose households did not directly benefit from the treatment [21]. Although these studies offer persuasive evidence of the economic recovery following ART initiation for those with low CD4 counts, no study to date assessed whether ART initiation among asymptomatic patients with CD4 counts above 500 cells/μl also generates economic benefits. Crucial to the discussion of economic benefits of “test and treat” is the hypothesis that delayed ART initiation may lead to a period of health and economic decline along with incomplete recovery even when patients adhere to ART. Thus, averting a decline of outcomes through early ART initiation may offer important benefits that extend beyond the health sector.

Although a positive association between employment outcomes and CD4 counts among those not on ART has previously been documented in a rural Ugandan setting [22] and a study from South Africa has shown that employment outcomes decline in the years prior to ART initiation [15], data from a larger population on a wider set of indicators of economic well-being are lacking. Furthermore, information on viral loads of HIV-positive persons has seldom been integrated into analyses. Because most studies have focused on outcomes of patients initiating ART, less is also known about how HIV-negative household members fare when an HIV-positive household member has high vs. low CD4 counts. This study utilizes detailed socio-economic and laboratory data collected as part of a community cluster randomized trial conducted in east Africa to examine the association between HIV status, CD4 counts, viral suppression and ART initiation, and multiple indications of economic well-being.

## Methods

### Study procedures

Data were collected in 32 rural communities participating in the ongoing Sustainable East Africa Research in Community Health (SEARCH) HIV “test and treat” cluster randomized controlled trial (NCT01864603). The trial procedures have been described in detail elsewhere [23]. Briefly, study communities consisting of approximately 10,000 individuals each are located in three distinct geographic regions with varying HIV prevalence; 12 communities are in western Kenya, 10 in eastern Uganda, and 10 in southwestern Uganda. At the beginning of the trial (baseline), a door-to-door census was conducted in each community and was followed by 2-week multi-disease community health campaigns (CHCs) that included HIV testing, counselling, and referral to care for HIV-positive persons. Individuals aged >10 years who participated in census but did not attend the CHCs were subsequently approached for home-based testing. This “hybrid” testing approach was found to achieve 89% population-level HIV testing coverage [23].

Following the CHCs and home-based testing, a random sample of households with and without an HIV-positive adult were selected for structured household socio-economic (HSE) surveys. The CHC campaigns usually took place 2 weeks prior to the start of HSE data collection. Sampling of household occurred after the door-to-door census and the ‘hybrid’ testing approach was completed in each community. These activities made it possible to assess HIV status of nearly all community members, which was then used for sampling purposes. For the HSE surveys, households with HIV-positive adults were over-sampled in each community given the likelihood that these households would be most directly affected by the primary intervention in the SEARCH trial (i.e. earlier initiation of HIV treatment). All households were classified as having at least one HIV-positive adult (≥18 years) or having no HIV-positive adults. In each community, we sampled 100 households that included an HIV-positive adult and 100 households that did not include an HIV-positive adult.

The HSE surveys, adapted from the World Bank’s Living Standards Measurement Surveys [24], sought to assess socio-economic conditions of households and individuals residing in them. They consisted of individuals’ demographic characteristics, employment and income of household members aged ≥12 years, ownership of durable goods and livestock, healthcare utilization, and education of household members aged 6–25 years. Reports about all household members were obtained from one adult, typically the household head or spouse of household head. Survey questionnaires were administered by trained research assistants who visited the households. Information collected in the household surveys was linked at the individual level to SEARCH data on HIV status, and for HIV-positive individuals, on CD4 count and viral load.

### Ethics statement

The Makerere University School of Medicine Research and Ethics Committee (Uganda), the Uganda National Council for Science and Technology (Uganda), the Kenya Medical Research Institute Scientific Ethics Review Unit (Kenya), and the University of California San Francisco Committee on Human Research (USA) approved the consent procedures and the study. All participants provided verbal informed consent in their preferred language with a signature or fingerprint confirmation of consent.

### Outcome measures

Participation in the labor force was assessed with a binary indicator of whether an individual spent any time working (both on- or off-farm) in the week prior to the survey. Since most households were primarily engaged in subsistence farming, we separately evaluated participation in the agriculture sector with a binary indicator of whether an individual did any farm work in the past week. Self-reported health of study participants was measured with an indicator of any illness episodes in the past month. An illness episode included any type of illness or injury, for example cough, cold, diarrhea, or injury due to an accident. The potential economic burden of illness was measured with an indicator of whether the participant lost time from usual activities (employment and domestic activities) due to illness in the past month. Healthcare utilization was measured as a binary indicator of any care sought or received last month, any health expenditures last month, and any hospitalizations in the past year. Healthcare expenditures included travel for medical care costs, inpatient and outpatient fees, medicines, laboratories, and other healthcare costs. The recall period for hospitalization was one year prior to interview given the rarity of such events.

### Laboratory-confirmed indicators

HIV status of individuals was determined by rapid HIV tests obtained in the hybrid testing approach, i.e. CHCs and home-based testing. Laboratory-confirmed CD4+ T-cell/μL counts measured at baseline CHCs and home-based testing were used to characterize HIV-positive participants’ disease stages [23]. HIV RNA levels were measured as previously described [25]; viral suppression was defined by RNA levels <500 copies/μL. Finally, we used ART start date, based on information extracted from Ministry of Health data and study records, to determine whether HIV-positive participants initiated ART at least 1 month prior to the household survey.

### Statistical analyses

We conducted two sets of complementary analyses. First, we focused on HIV-positive persons and examined the association between HIV status, HIV disease progression, and socio-economic outcomes. We compared outcomes of HIV-positive participants grouped by their CD4 counts to outcomes of HIV-negative participants in households without an HIV-positive person (a group of individuals that would be least-affected by HIV/AIDS). HIV-positive participants were grouped by their CD4 counts in three categories that reflect various thresholds used for ART initiation: >500 cells/μL, 351–500 cells/μL, and ≤350 cells/μL.

Second, we focused on HIV-negative persons and sought to examine the association between having an HIV-positive household member and various socio-economic outcomes. We therefore compared outcomes of HIV-negative participants grouped by their HIV-positive household members’ CD4 counts to the outcomes of HIV-negative participants in households without any HIV-positive person. HIV-negative participants in households with an HIV-positive person were grouped based on the HIV-positive person’s CD4 count. In cases where a household had ≥2 HIV-positive persons, the lowest CD4 count was used to categorize participants. In all models, HIV-negative participants who lived in households without any HIV-positive person served as the reference group.

We estimated the following regression models:

$$Y_{ijk}=HIV\_CD4{’}_{ijk}\;\mu +ART_{ijk}\;\beta +X{’}_{ijt}\;\varphi +\gamma_{k}+\varepsilon_{ijk}$$ (1)

where *i* indexed individual living in household *j* in community *k*. *HIV_CD4* was a vector of HIV status and CD4 count categories, *ART* indicated whether the individual had initiated ART at least one month prior to the interview, and *X* was a vector of individual- and household-level characteristics: age, age-squared (to capture non-linear effects of age), education (indicators for no education, some primary education, completed primary education, some secondary education, and completed secondary education or greater), gender (indicator for female), marital status (indicator for married or cohabiting), wealth index (in quintiles), number of children in household, and household size. ART status was included as a control variable in order to allow for the possibility that individuals with similar CD4 counts might have different outcomes depending on whether they were on ART. In order to retain HIV-negative participants in the model as the reference group, they were coded as “not on ART”. Community fixed effects, *γ*_*k*_, were included to control for underlying differences between the study communities. Wealth index was calculated using the first component of a principal component analysis of ownership indicators of 47 different household items [26].

We used generalized estimating equations (GEE) regression models to analyze binary dependent variables as a function of HIV status and HIV disease stages, defined by laboratory-confirmed CD4 T-cell counts. We fitted GEE models using binomial distribution, logistic link, and exchangeable correlation at the household level [27]. Given the difficulty of interpreting logistic regression coefficients (log odds) and exponentiated coefficients (odds ratios), we instead report average marginal effects (ME). Marginal effects represent the percentage point change in predicted probability of the outcome for a one unit increase of the independent variable (or percentage point change from base for discrete variables), holding all other variables constant. We restricted the analytic sample to individuals who had non-missing data for all outcomes and explanatory variables of interest. Post estimation Wald tests were used to perform two-sided hypothesis tests about equality of the CD4>500 coefficient to the CD4 351–500 and CD4 ≤350 coefficients (and the equivalent of HIV-negative participant groups based on HIV-positive household members’ CD4 counts).

We also tested whether there was an association between CD4 counts and economic outcomes among HIV-positive participants based on their ART and viral suppression status. This GEE estimation sub-categorized HIV-positive participants in the 3 CD4 groups by whether they initiated ART and whether they reached viral suppression. High CD4 participants who were virally suppressed were assumed to have achieved recovery in health status through ART. High CD4 participants who were not virally suppressed were assumed to include newly infected HIV participants identified through the comprehensive SEARCH testing strategy, as described above, and patients who have been on ART but have not reached viral suppression. We also performed Wald tests to determine whether the association for those with CD4>500 not on ART was the same as for those with CD4>500 on ART and CD4 351–500 CD4 not on ART. Similar tests were performed with the viral suppression indicators.

Finally, we used nonparametric kernel-weighted local polynomial regression (with Epanechnikov kernels and zero degree polynomials for local mean smoothing) to visually examine the association between CD4 counts and study outcomes.

## Results

### Study participants

Data for 34,029 individuals living in 6,309 households were collected between July 2013 and August 2014. This sample represented 10.2% of the 335,024 residents in the SEARCH trial communities at baseline. We limited our analytic sample to working-age adults, defined as those aged 18–65 years (n = 14,449) whose HIV status was known (n = 11,873) and whose CD4 laboratory results were available if HIV-positive (n = 11,705). Participants with missing HIV test results were primarily male (63%), unmarried (53%), and young (50% aged <25 years and 75% aged <35 years). The final analytic sample was further restricted to participants who had non-missing data on outcomes and explanatory variables (n = 11,500 from 5,884 households).

Table 1 summarizes outcomes and other descriptive characteristics of the working-age adults in our sample with non-missing data. By design, nearly half the sample (48%) consisted of HIV-negative participants in households with no HIV-positive persons. Twenty-one percent of participants were HIV-negative and lived in households with ≥1 HIV-positive person. Approximately 31% of all participants (n = 3,626) were HIV-positive, and 50% (n = 1,798) of those individuals had CD4>500, 24% (n = 887) had CD4 351–500, and 26% (n = 941) had CD4≤350.

**Table 1:** Descriptive characteristics of study participants.

	Study Group					
	HIV-, no HIV+ household members	HIV-, lives with HIV+ household members	HIV+ CD4 >500	HIV+ CD4 351–500	HIV+ CD4 ≤350	Full Sample
No. individuals in study group	5,556	2,318	1,798	887	941	11,500
Percent of full sample in study group	48.3	20.2	15.6	7.7	8.2	100
*Study outcomes*						
Worked last week, %	70.9	58.8	79.2	75.8	74.3	70.4
Worked on a farm last week, %	58.7	49.2	63.1	62.3	57.4	57.7
Experienced illness last month, %	28.2	22.1	37.3	42.6	43.6	30.8
Lost time from usual employment or household activities due to illness last month, %	17.1	12.8	24.1	27.2	28.7	19.1
Sought or received care last month, %	16.7	13.5	25.6	30.0	31.3	19.7
Spent money on healthcare last month, %	17.3	13.8	24.9	29.8	29.4	19.8
Hospitalized last year, %	5.6	4.5	9.3	10.9	13.5	7.0
*Descriptive characteristics*						
Enrolled in ART, %	0.0	0.0	42.6	47.2	49.4	14.3
Age, mean	34.3	32.0	36.2	38.5	39.2	34.9
Female, %	55.8	51.6	73.9	60.3	53.0	57.9
Married or cohabiting, %	68.4	53.6	67.7	67.8	69.0	65.3
No education, %	13.0	12.4	14.7	13.9	13.9	13.3
Some primary education, %	41.5	38.7	47.6	47.9	45.1	42.7
Completed primary, %	16.6	14.3	18.8	18.7	20.0	16.9
Some secondary, %	18.1	23.4	13.1	13.5	13.6	17.7
Completed secondary or higher, %	10.8	11.2	5.8	6.0	7.4	9.5
Wealth quintile, mean	3.2	3.4	3.0	2.9	3.0	3.2
Number of children, mean	3.5	3.5	3.0	2.9	2.9	3.3
Household size, mean	6.5	7.2	5.4	5.3	5.4	6.3

**Abbreviations:** No., Number; %, Percent; HIV+, HIV-positive; HIV-, HIV-negative; ART, antiretroviral therapy.

**Notes:** HIV-negative participants are stratified based on whether they resided with HIV-positive household member. Antiretroviral therapy status at baseline was established using first medication pickup date. Participants were defined as being enrolled in ART if their first ART pickup date was before the CHC campaign began in their community. Wealth, in quintiles, was calculated using Principal Components Analysis and is based on ownership of 47 items.

More than half of participants (56%) had completed less than primary education, with HIV-negative individuals having higher education and slightly higher household wealth. The average age among participants was 35 years and nearly 58% were women. Majority of participants were active in the labor force in the past week, with 70% and 58% of all participants reporting having done any work and farm work, respectively. Labor force participation rates were lowest for HIV-negative participants in households with ≥1 HIV-positive person. Approximately one-third of all participants reported an illness in the past month and nearly one-fifth of all participants reported having sought or received healthcare in the past month, having lost time from usual activities due to illness, and having spent money on treatment in the past month. Hospitalization in the previous year was reported by 7% of all participants, with HIV-positive participants being most likely to be hospitalized. Overall, HIV-positive participants reported more illness and higher healthcare expenditures.

### Regression results

**Fig 1:**
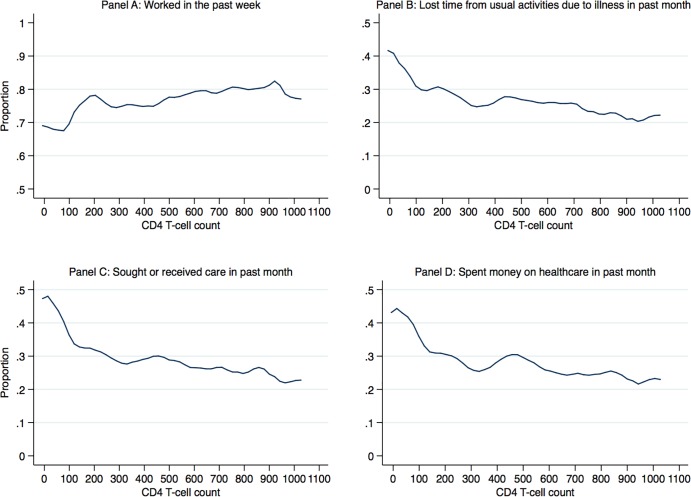
Local polynomial graphs of employment and healthcare outcomes and CD4 counts among HIV-positive adults.

Fig 1 displays the results of nonparametric regressions that estimated the unadjusted association between CD4 counts and four indicators of employment and healthcare utilization outcomes among HIV-positive adults. HIV-positive adults with higher CD4 counts had higher probability of work last week, lower probability of losing time from usual activities due to illness, lower probability of spending time seeking healthcare or losing time from usual activities due to illness. Generally, the largest changes in probability of outcomes were associated with the lowest CD4 counts, below 350 cells, at which point the probability continued to change but at a lower rate.

#### Employment outcomes of HIV-positive adults

Results in Table 2 show the association of health status of HIV-positive participants, as indicated by CD4 counts, with employment outcomes. Compared to the reference group of HIV-negative participants who did not reside with an HIV-positive person, HIV-positive participants with CD4>500 had equal probability of working in the past week as the reference group (marginal effect, ME 1.49; 95% confidence interval, CI: -1.09, 4.08). HIV-positive participants with CD4 351–500, on the other hand, were significantly less likely to have worked in the past week (ME -4.50; 95% CI -7.99, -1.01) as were participants with CD4≤350 (ME -7.41; 95% CI -10.96, -3.85). Post-estimation Wald tests showed HIV-positive participants with CD4>500 were more likely to have worked than participants with CD4 351–500 and CD4 ≤350 (*p*<0.001 in both tests). We found largely similar patterns when looking specifically at farm work, with HIV-positive participants with high CD4 counts being similar to the reference group and those with low CD4 counts less likely to be doing farm work. Being on ART was associated with higher probability of having done any work in the past week (ME 3.34; 95% CI 0.59, 6.10) as well as any farm work (ME 4.65; 95% CI 1.60, 7.70).

**Table 2:** Employment of HIV-positive working-age adults compared to HIV-negative adults living in households with no infected household members.

	Any work last week		Any farm work last week	
	ME	[95% CI]	ME	[95% CI]
*HIV-*, *no HIV+ household member*	*Ref*		*Ref*	
HIV+, CD4 >500	1.49	[-1.09, 4.08]	-2.20	[-5.12, 0.73]
HIV+, CD4 351–500	-4.50*	[-7.99, -1.01]	-3.78*	[-7.50, -0.07]
HIV+, CD4 ≤ 350	-7.41***	[-10.96, -3.85]	-9.52***	[-13.22, -5.81]
*Not on ART*	*Ref*		*Ref*	
On ART	3.34*	[0.59, 6.10]	4.65**	[1.60, 7.70]
Mean outcome for reference group	70.9		58.7	
No. observations	9,182		9,182	
*P-values from post-estimation Wald tests*				
HIV+, CD4 >500 = HIV+, CD4 351–500	<0.001		0.402	
HIV+, CD4 >500 = HIV+, CD4 ≤350	<0.001		<0.001	

**Abbreviations:** ME, marginal effect; 95% CI, 95% confidence interval, HIV+, HIV-positive; HIV-, HIV-negative; ART, antiretroviral therapy.**Notes:** GEE regression models for working age adults (18–65 years old), with binomial distribution, logistic link, and exchangeable correlation at the household level. Marginal effects are reported as percentage point changes. Reference group are HIV-negative participants who do not reside with HIV-positive household members. Models controlled for education level, age, age-squared, gender, marital status, wealth index, number of children in household, number of household members and community indicators. P-value notation

*** p<0.001

** p<0.01

* p<0.05

#### Employment outcomes of HIV-negative adults

Among HIV-negative participants, those residing in households with an HIV-positive person who had low CD4 counts (≤350 or 351–500) were less likely to be engaged in the labor force than the reference group (Table 3). For example, those in households with an HIV-positive person with CD4≤350 were 6.28 percentage points less likely to have worked in the past week (95% CI -10.75, -1.80) and 4.89 percentage points less likely to have done farm work in the past week (95% CI -9.74, -0.03). In contrast, HIV-negative participants in household with an HIV-positive person with CD4>500 had statistically equal probability of working (ME -1.78; 95% CI -5.16, 1.59) as their counterparts in households with no HIV-positive persons. Post-estimation Wald tests showed that probability of any work and farm work last week were statistically higher for individuals whose household members had >500 CD4 counts than individuals whose household members had CD4 351–500 (*p =* 0.029 and *p =* 0.019, respectively).

**Table 3:** Employment of HIV-negative working-age adults who have HIV-positive household members compared to HIV-negative adults living in households with no infected household members.

	Any work last week		Any farm work last week	
	ME	[95% CI]	ME	[95% CI]
*HIV-*, *no HIV+ household member*	*Ref*.		*Ref*.	
HIV-, lives with HIV+ CD4 >500	-1.78	[-5.16, 1.59]	-0.84	[-4.56, 2.88]
HIV-, lives with HIV+ CD4 351–500	-7.03**	[-11.49, -2.57]	-7.00**	[-11.79, -2.21]
HIV-, lives with HIV+ CD4 ≤350	-6.28**	[-10.76, -1.80]	-4.89*	[-9.74, -0.03]
*No household member on ART*	*Ref*.		*Ref*.	
All HIV+ household members on ART	0.35	[-3.47, 4.17]	0.00	[-4.35, 4.36]
Some HIV+ household members on ART	2.60	[-5.97, 11.17]	1.76	[-8.56, 12.08]
Mean outcome for reference group	70.9		58.7	
No. observations	7,874		7,874	
*P-values from post-estimation Wald tests *				
HIV-, CD4 >500 = HIV-, CD4 351–500	0.029		0.019	
HIV-, CD4 >500 = HIV-, CD4 ≤350	0.056		0.117	

**Abbreviations:** ME, marginal effect; 95% CI, 95% confidence interval, HIV+, HIV-positive; HIV-, HIV-negative; ART, antiretroviral therapy.**Notes:** GEE regression models for working age adults (18–65 years old), with binomial distribution, logistic link, and exchangeable correlation at the household level. Marginal effects are reported as percentage point changes. Reference group are HIV-negative participants who do not reside with HIV-positive household members. Models controlled for education level, age, age-squared, gender, marital status, wealth index, number of children in household, number of household members, and community indicators. P-value notation

*** p<0.001

** p<0.01

* p<0.05

#### Health and healthcare utilization of HIV-positive adults

HIV-positive individuals in all CD4 categories were significantly more likely to have experienced illness, lost time from usual activities due to illness, sought healthcare, spent money on healthcare and to have been hospitalized than HIV-negative participants in households with no HIV-negative persons (Table 4). For example, compared to the reference group, probability of seeking healthcare in the past month was much higher for HIV-positive participants with CD4>500 (ME 4.21; 95% CI 1.75, 6.66), CD4 between 351–500 (ME 8.46; 95% CI 5.15, 11.76), and CD4≤350 (ME 9.63; 95% CI 6.31, 12.94). Participants with CD4≤350 were 6.75 percentage points more likely to have been hospitalized in the past year than the reference group (95% CI 4.27, 9.22). In general, HIV-positive participants with CD4≤350 and CD4 351–500 had similar healthcare utilization levels and participants with CD4>500 had lower levels. However, the likelihood of hospitalization in the past 12 months was significantly higher among those with CD4<350 than any other study group.

**Table 4:** Illness and health seeking behaviors of HIV-positive working-age adults compared to HIV-negative adults living in households with no infected household members.

	Experienced illness last month		Lost time from usual activities due to illness last month ^a^		Sought or received care last month		Spent money on healthcare last month		Hospitalized last year ^b^	
	ME	[95%CI]	ME	[95%CI]	ME	[95%CI]	ME	[95%CI]	ME	[95%CI]
*HIV-*, *no HIV+ household member*	*Ref*.		*Ref*.		*Ref*.		*Ref*.		*Ref*.	
HIV+, CD4 >500	3.22*	[0.42, 6.03]	3.24**	[0.81, 5.67]	4.21***	[1.75, 6.66]	3.68**	[1.21, 6.15]	2.04*	[0.48, 3.60]
HIV+, CD4 351–500	8.72***	[5.05, 12.39]	6.20***	[2.98, 9.42]	8.46***	[5.15, 11.76]	8.57***	[5.23, 11.91]	3.95***	[1.72, 6.17]
HIV+, CD4 ≤350	9.80***	[6.15, 13.46]	7.42***	[4.19, 10.66]	9.63***	[6.31, 12.94]	7.93***	[4.65, 11.22]	6.75***	[4.27, 9.22]
*Not on ART*	*Ref*.		*Ref*.		*Ref*.		*Ref*.		*Ref*.	
On ART	1.68	[-1.27, 4.63]	1.58	[-0.97, 4.12]	2.99*	[0.40, 5.59]	2.04	[-0.55, 4.62]	1.22	[-0.43, 2.86]
Mean outcome for reference group	28.2		17.1		16.7		17.3		5.6	
No. observations	9,182		9,182		9,182		9,182		9,182	
*P-values for post-estimation Wald tests *										
HIV+, CD4 >500 = HIV+, CD4 351–500	0.003		0.072		0.011		0.004		0.094	
HIV+, CD4 >500 = HIV+, CD4 ≤350	<0.001		0.011		0.001		0.011		<0.001	

**Abbreviations:** ME, marginal effect; CI, confidence interval; HIV+, HIV-positive; HIV-, HIV-negative; ART, antiretroviral therapy

**Notes:** GEE regression models for working age adults (18–65 years old), with binomial distribution, logistic link, and exchangeable correlation at the household level. Marginal effects are reported in percent changes. Reference group are HIV-negative participants who do not reside with HIV-positive household members. Models controlled for education level, age, age-squared, gender, marital status, wealth index, number of children in household, number of household members, and community indicators. P-value notation

*** p<0.001

** p<0.01

* p<0.05

^**a**^ Usual activities include paid and unpaid work such as household chores.

^**b**^ Hospitalizations captured using one year recall period.

#### Healthcare utilization of HIV-negative adults

HIV-negative participants who lived with HIV-positive household members were less likely to lose time from usual activities due to own illness or care-seeking, and spend money on healthcare than their counterparts in households without an HIV-positive person (Table 5). For example, HIV-negative participants whose household members had CD4 counts in the 351–500 range were 4.53 percentage points less likely to lose time from activities due to illness in the past month (95% CI -7.82, -1.23), 3.31 percentage points less likely to seek or receive healthcare in the past month (95% CI -6.66, 0.03), and 3.85 percentage points less likely to spend money on healthcare (95% CI -7.25, -0.45).

**Table 5:** Illness and health seeking behaviors of HIV-negative working-age adults who have HIV-positive household members compared to HIV-negative adults living in households with no infected household members.

	Lost time from usual activities due to illness last month ^a^		Sought or received care last month		Spent money on healthcare last month		Hospitalized last year ^b^	
	ME	[95%CI]	ME	[95%CI]	ME	[95%CI]	ME	[95%CI]
*HIV-*, *no HIV+ household member*	*Ref*.		*Ref*.		*Ref*.		*Ref*.	
HIV-, lives with HIV+ CD4 >500	-1.74	[-4.58, 1.09]	-0.85	[-3.68, 1.98]	-1.18	[-4.08, 1.72]	-0.14	[-1.85, 1.58]
HIV-, lives with HIV+ CD4 351–500	-4.53**	[-7.82, -1.23]	-3.31	[-6.66, 0.03]	-3.85*	[-7.25, -0.45]	-0.36	[-2.54, 1.82]
HIV-, lives with HIV+ CD4 ≤350	-3.30	[-6.74, 0.13]	-0.88	[-4.46, 2.71]	-1.86	[-5.45, 1.74]	-0.53	[-2.63, 1.57]
*No household member on ART*	*Ref*.		*Ref*.		*Ref*.		*Ref*.	
All HIV+ household members on ART	3.15	[-0.70, 7.01]	1.74	[-1.89, 5.37]	2.42	[-1.36, 6.20]	-0.29	[-2.30, 1.72]
Some HIV+ household members on ART	-1.01	[-10.08, 8.07]	-2.42	[-10.89, 6.05]	-3.01	[-11.43, 5.40]	-2.95	[-6.39, 0.48]
Mean outcome for reference group	17.1		16.7		17.3		5.6	
No. observations	7,874		7,874		7,874		7,874	
*P-values for post-estimation Wald tests *								
HIV-, CD4 >500 = HIV-, CD4 351–500	0.129		0.191		0.161		0.856	
HIV-, CD4 >500 = HIV-, CD4 ≤350	0.394		0.991		0.725		0.732	

**Abbreviations:** ME, marginal effect; 95% CI, 95% confidence interval, HIV+, HIV-positive; HIV-, HIV-negative; ART, antiretroviral therapy.

**Notes:** GEE regression models for working age adults (18–65 years old), with binomial distribution, logistic link, and exchangeable correlation at the household level. Marginal effects are reported in as percentage point changes. Reference group are HIV-negative participants who do not reside with HIV-positive household members. Models controlled for education level, age, age-squared, gender, marital status, wealth index, number of children in household, number of household members, and community indicators. P-value notation

*** p<0.001

** p<0.01

* p<0.05

^**a**^ Usual activities include paid and unpaid work such as household chores.

^**b**^ Hospitalizations captured using one year recall period.

#### Associations based on ART status and viral load suppression

**Fig 2:**
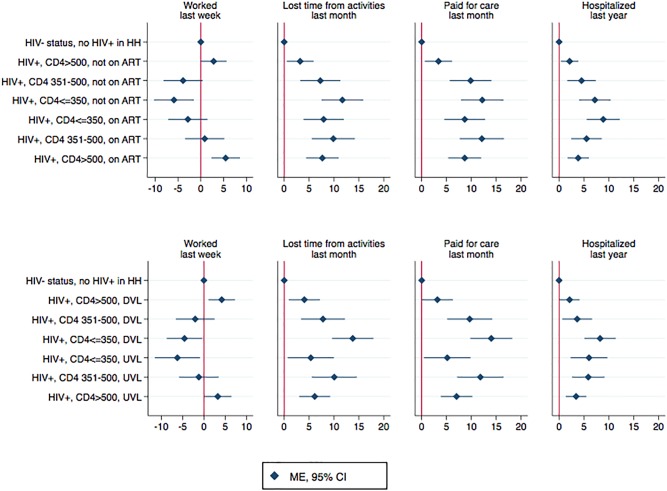
Association between CD4 count and socio-economic outcomes, based on ART status and viral load. **Abbreviations:** ART, antiretroviral therapy; CI, confidence interval; DVL, detectable viral load, HIV+, HIV-positive; HIV-, HIV-negative; HH, household; ME, marginal effect. **Notes:** Marginal effects, in percentage points, with 95% confidence intervals around them, were calculated after fitting GEE regression models for working age adults (18–65 years old), with binomial distribution, logistic link, and exchangeable correlation at the household level. Reference group were HIV-negative participants in households that did not have any HIV-positive adults. Models controlled for education level, age, age-squared, gender, marital status, wealth index, number of children in household, number of household members, and community indicators.

Finally, Fig 2 presents findings from models where HIV-positive participants were sub-divided in CD4 groups based on whether they had initiated ART and had achieved viral suppression. The subcategories are presented in the order of how we conceptually assumed people transition through HIV disease stages (i.e. declining CD4 counts for people not on ART or with detectable viral load and improving CD4 counts for people on ART or with suppressed viral load). As can be seen from the figures, declining CD4 counts were associated with progressively worse outcomes, with the lowest probability of employment and highest probability of healthcare utilization and expenditures found among those in with CD4<350. Higher CD4 counts among participants who were on ART or who had achieved viral suppression was generally associated with better economic outcomes. Notably, high CD4 participants not on ART who had detectable viral loads (i.e. newly identified cases) were found to have similar probability of outcomes as the HIV-negative participants. HIV-positive participants whose CD4 counts recovered to CD4>500 reported economic losses due to illness that were higher compared to both HIV-negative and newly identified HIV-positive participants with high CD4.

## Discussion

In this large, population-representative study from 32 communities participating in the SEARCH trial in Kenya and Uganda, economic outcomes for HIV-positive adults with CD4 counts above 500 were significantly better than those for HIV-positive adults with CD4 counts below 500, and also statistically equivalent to outcomes for HIV-negative adults. For HIV-negative household members of HIV-positive persons, higher CD4 count of an HIV-positive household member was similarly protective of employment outcomes. Patterns in healthcare utilization and healthcare costs incurred by HIV-positive individuals also indicated a benefit to having higher CD4 counts, although the outcomes of those with CD4>500 remained worse than those for HIV-negative persons in households not directly affected by HIV. Importantly, even among HIV-positive participants who had not initiated ART or were not virally suppressed, those with CD4>500 had better economic outcomes than those with lower CD4 counts, including in the mid-range of 351–500 cells/μL. These findings suggest that initiating ART before CD4 counts decline substantially could generate economic benefits, though the results need to be confirmed with longitudinal data to establish causality.

Prior population-based studies of adults initiating ART have often lacked data on key health indicators such as the CD4 count and viral load of individuals, have relied on clinic-based samples that only included those who seek care, or have studied HIV-positive individuals in settings where ART initiation typically did not occur until CD4 counts declined to threshold levels such as 200, as specified in previous guidelines [10–17, 28]. Our study offers a more generalizable depiction of the association between CD4 counts and economic outcomes in the general population. Although some previous studies have documented an individual-level decline in employment outcomes in the years prior to ART initiation, followed by a rebound in economic outcomes after initiating treatment at low CD4 counts [15, 29, 30], studies that assess outcomes of large numbers of asymptomatic, high CD4 adults are lacking. Thus, our study fills a gap in literature by including a large, population-representative sample that reflects the communities that would benefit from “test and treat”. By and large, the findings from our cross-sectional study are consistent with the hypothesis that population-level HIV testing and early ART initiation may help avert an economic decline associated with HIV disease progression.

Previous studies document that early ART initiation offers important individual health benefits and reduces the risk of HIV transmission [1, 2]. Our study findings provide policymakers with additional data on the potential economic gains of finding and treating persons with HIV early in their disease course. It is particularly noteworthy that HIV-positive adults with CD4>500 generally had significantly higher employment rates than even those with CD4 cell counts between 351–500. It is important to recognize that our study precludes causal inference and that further evidence from longitudinal assessments are needed to determine the causal impact of early ART on economic outcomes. The associations we found do suggest, however, that the benefits of early ART initiation may be sizable when it comes to outcomes such as employment, healthcare utilization and expenditures.

This study is also noteworthy for its analysis of how HIV-negative household members are affected by the health status on an HIV-positive person. The findings suggest several important “spillover effects” of early HIV treatment. In households where HIV-positive adults had high CD4 counts, HIV-negative household members typically had similar employment outcomes as HIV-negative individuals in households without an HIV-positive person. This was not the case in households where HIV-positive adults had CD4≤500. This finding suggests that maintaining high CD4 counts can have population-level economic impacts that are greater than those among HIV-positive persons alone. Consistent with this possibility, it is notable that a recent study showed that the introduction of PEPFAR in SSA, which led to many HIV-positive persons with low CD4 counts initiating ART, was associated with an increase in population-level employment outcomes [17]. Another study found that proximity to ART increased work hours of HIV-negative people whose households did not directly benefit from expanded treatment [21]. Our study adds to the evidence indicating that ART may have spillover effects that extend beyond the HIV-positive persons receiving treatment.

Our findings also suggest that in households faced with high costs of care and treatment of an HIV-positive person, other household members may be forgoing some of their routine healthcare needs. This could have important future repercussions if untreated diseases of HIV-negative individuals lead to worsening symptoms, costlier treatment, and exit from the labor market. Thus, early access to ART may offer important cost savings at the population-level in the long term. If these trends are confirmed in prospective analyses, future cost-effectiveness studies should consider incorporating in their models the broader spillover effects of early ART that we report here rather than relying solely on the individual costs and benefits for the treated person [7].

Our findings have greater relevance given the recent focus on achieving 90-90-90 targets through greatly expanded HIV testing and access to ART, the emphasis on the need for increased resources to do this [6], as well as the accompanying concern that development assistance for health has stagnated and future funding levels are uncertain [31]. The increased demands on countries financing their own programs as well as the proposed cuts to U.S. foreign assistance [9] could undermine prospects of achieving the 90-90-90 targets in countries that are most affected by HIV/AIDS. Along with a series of other economic studies, this analysis provides compelling evidence of the economic and health system benefits that are associated with protecting and improving the health outcomes of HIV-positive persons. Evidence presented in this study can be useful for governments of low-income countries for priority-setting in their budgets and for US policy makers for decisions about future funding levels of foreign aid.

Key strengths of the paper include evaluating multiple socio-economic outcomes in large, population-representative sample that included HIV-positive participants and their household members. Access to laboratory-confirmed HIV status and CD4 counts allowed us to assess the health status of study participants using objective measures. We measured important, yet seldom evaluated, indirect costs associated with ART. Despite ART drugs being offered free of charge to eligible patients in SSA, studies have documented significant costs associated with being on ART, including drugs to treat opportunistic infections or comorbidities, non-routine laboratory tests, medical consultations, and hospital stays [32–34]. We found that HIV-positive participants in all CD4 count categories reported more interactions with the healthcare system, though high CD4 participants fared better than those with low CD4 counts. Some results from this study also confirmed and extended previous findings about the associations between severity of HIV disease, ART, and employment outcomes. Consistent with existing literature, we found ART initiation was associated with better economic outcomes [10, 12, 19, 22, 28]. The findings for individuals with CD4 counts >500 confirmed, with greater precision, the results from a small pilot study that our team conducted in one community in southwestern Uganda [22].

Our study has several notable limitations. First, the study does not establish a causal relationship between the main health status measures (HIV status, CD4 count, and viral suppression status) and economic outcomes. In particular, the associations reported here may be biased by uncontrolled confounding. One concern is selection bias stemming from the possibility that HIV-positive individuals who sought treatment earlier may be more likely to benefit from ART or have better underlying economic circumstances. Another concern is the possibility of reverse causality, with individuals who have better economic characteristics being more likely to engage in care and achieve better health outcomes. Nonetheless, the overall consistency of the main findings with other studies showing declining health in the years prior to ART initiation suggests that such biases are not a major concern.

The study conclusions are further limited by the fact that HIV status was missing for more than 2,500 (or 18%) of working-age adults who were part of the HSE survey and who were deemed to be stable residents at census. We compared descriptive characteristics and outcomes of participants with missing HIV status and found that these participants were more likely to be younger, single, male, and in school and were less likely to be working, experience illness, lose time from work due to illness or use/pay for healthcare than our study participants. Although participants with missing HIV status were more similar to HIV-negative participants than HIV-positive participants in our sample, we can only speculate about their actual HIV status and disease stage if HIV positive. If the participants with missing HIV status had lower HIV prevalence than those not missing HIV status, then including them in our sample would have magnified our study findings. If these participants had higher HIV prevalence and lower CD4 counts than what was observed among those whose HIV status was measured, our findings would also be underestimates. On the other hand, if the participants with missing HIV status had a higher proportion of high CD4, HIV-positive adults, our findings would have overestimated the association between high CD4 counts and economic outcomes. Follow-up longitudinal data collection in the SEARCH trial is ongoing and we will be able to estimate the causal effect of early ART initiation on various economic outcomes using prospective data once the trial completes.

Another limitation is measurement bias. Many outcome measures were obtained in household surveys and could have suffered from recall bias. We attempted to limit these biases by using short recall periods that are common in similar literature [35]. Moreover, the main indicators of health status were laboratory-confirmed measures of HIV status and CD4 counts. The use of objective measures of health status represents an advance in comparison to previous studies that examined the link between health and economic outcomes, which relied on self-reported health status [36]. ART status was based on treatment initiation dates rather than adherence; this could have led to misclassification of some people as being on ART (and therefore having better health) even if they did not have high adherence. For this reason, it is possible that our estimates of the association between ART status and economic outcomes were biased downward. Finally, the employment outcomes we relied on in this study were measures of labor market participation rather than productivity. Future studies should incorporate more robust measures of labor productivity to improve our understanding of how HIV impacts the earning potential of people living with the disease.

## Conclusion

At a time when countries most affected by the HIV epidemic face increasing financial challenges to fund the HIV response, our study provides evidence about the potential economic benefits of earlier ART initiation. While we cannot draw causal conclusions, our findings provide important insights about the associations between the CD4 counts and ART use of HIV-positive persons and the economic outcomes they and their HIV-negative household members achieve. Future data from the SEARCH trial will enable us to test the hypothesis that early ART initiation has a causal effect on individual- and household-level economic outcomes.

## Supporting information

S1 File SEARCH baseline HSE replication dataset. (DTA)
